# Acromegaly: Clinical Care in Central and Eastern Europe, Israel, and Kazakhstan

**DOI:** 10.3389/fendo.2022.816426

**Published:** 2022-02-22

**Authors:** Marek Bolanowski, Zaina Adnan, Mirjana Doknic, Mykola Guk, Václav Hána, Irena Ilovayskaya, Darko Kastelan, Tomaz Kocjan, Martin Kužma, Akmaral Nurbekova, Catalina Poiana, Nikolette Szücs, Silvia Vandeva, Roy Gomez, Sorin Paidac, Damien Simoneau, Ilan Shimon

**Affiliations:** ^1^ Department of Endocrinology, Diabetes and Isotope Therapy, Wroclaw Medical University, Wroclaw, Poland; ^2^ Division of Endocrinology and Metabolism, Bar Ilan Faculty of Medicine, Clalit Medical Health Care Services, Safed, Israel; ^3^ Neuroendocrinology Department, Clinic for Endocrinology, Diabetes and Metabolic Diseases, Clinical Centre of Serbia, Medical Faculty University of Belgrade, Belgrade, Serbia; ^4^ Department of Transsphenoidal Surgery, Romodanov Neurosurgery Institute, Kyiv, Ukraine; ^5^ Third Department of Internal Medicine, First Faculty of Medicine, Charles University and General University Hospital, Prague, Czechia; ^6^ Neuroendocrine Unit, Endocrinology Department, Moscow Regional Research and Clinical Institute, Moscow, Russia; ^7^ Department of Endocrinology, University Hospital Center Zagreb, Zagreb, Croatia; ^8^ Department of Endocrinology, Diabetes and Metabolic Diseases, University Medical Centre Ljubljana, Ljubljana, Slovenia; ^9^ Faculty of Medicine, University of Ljubljana, Ljubljana, Slovenia; ^10^ Comenius University Faculty of Medicine, 5th Department of Internal Medicine, University Hospital Bratislava, Bratislava, Slovakia; ^11^ Kazakh National Medical University, Almaty, Kazakhstan; ^12^ Department of Endocrinology, “Carol Davila” University of Medicine and Pharmacy, Bucharest, Romania; ^13^ Department of Internal Medicine and Oncology, Semmelweis University, Budapest, Hungary; ^14^ Department of Endocrinology, Specialized Hospital for Active Treatment of Endocrinology, Medical University, Sofia, Bulgaria; ^15^ Global Medical Affairs, Pfizer Rare Disease, Brussels, Belgium; ^16^ Medical Affairs, Pfizer Rare Disease, Bucharest, Romania; ^17^ Central and Eastern Europe Medical Affairs, Pfizer Rare Disease, Paris, France; ^18^ Rabin Medical Center, Petah-Tikva, and Sackler Faculty of Medicine, Tel-Aviv University, Tel-Aviv, Israel

**Keywords:** acromegaly, IGF-I, referral pathway, endocrinology, multidisciplinary care

## Abstract

Acromegaly is a rare condition typically caused by benign pituitary adenomas, resulting in excessive production of growth hormone. Clinical manifestations of acromegaly are diverse, varying from the overgrowth of body tissue to cardiovascular, metabolic, and osteoarticular disorders. Symptoms may emerge slowly, overlapping with other diseases and often involve many different healthcare specialists. In the last decade, efforts to provide an accurate and timely diagnosis of acromegaly have improved disease management and clinical experience. Despite this progress, marked differences in the diagnosis, treatment, and management of acromegaly exist from country-to-country. To address these inconsistencies in the region comprising Central and Eastern Europe, Israel, and Kazakhstan, a panel of acromegaly experts from 13 of these countries was convened. Acromegaly experts from each country provided available information on the approaches from their country, including regional treatment centers and multidisciplinary teams, treatment access, reimbursement and availability, and physician education, disease awareness, and patient advocacy. Across several areas of acromegaly management, divergent approaches were identified and discussed, including the provision of multidisciplinary care, approved and available treatments, and disease awareness programs. These were recognized as areas of potential improvement in the management of acromegaly, in addition to participation in national and regional acromegaly registries. Further experience exchange will facilitate the identification of specific strategies that can be adapted in each country, and widespread participation in acromegaly registries will enable their evaluation. It is anticipated that this approach will support the optimization of acromegaly patient care across this region.

## Introduction

Acromegaly is a rare disease characterized by excess growth hormone (GH) secretion, which promotes the increased synthesis of insulin-like growth factor-I (IGF-I). Benign pituitary adenomas represent the leading cause of acromegaly, and in ~75% of cases, the excess GH production is driven by a pituitary macroadenoma (1). Clinical manifestations range broadly and may be related to the adenoma tumor mass itself, such as headache and visual impairment; or may be due to downstream effects of GH and IGF-I excess, for example, acral manifestations, respiratory disorders (such as snoring and sleep apnea), cardiovascular-related diseases, metabolic disorders (such as diabetes), and musculoskeletal disorders including osteoporosis, vertebral fractures and arthropathy (2). The increased mortality observed in patients with uncontrolled disease may be related in part to these disease-related comorbidities (3). A recently published large study involving 3173 patients with acromegaly from 10 European countries, including Bulgaria and the Czech Republic, reported that symptoms typically appear when patients are in their mid-thirties (median age: 33.5 years) (4). According to pooled data from 19 national acromegaly registries the mean age at diagnosis is about 45 years with an equal male to female ratio (1, 4).

## Acromegaly in Central and Eastern Europe, Israel & Kazakhstan

Taking into consideration the disease rarity, it is not uncommon for endocrinologists – even those in specialist pituitary centers – to have relatively small numbers (dozens) of acromegalic patients under their supervision (4). Therefore, expert meetings represent a valuable platform for exchanging experience and improving clinical assessment and management.

To promote the exchange of clinical experience and best practice within and across the Central and Eastern European region (CEE), Israel, and Kazakhstan, a panel of 14 experts (13 endocrinologists and one neurosurgeon) from the region was convened to exchange insights on the diagnosis, treatment and follow-up of acromegaly. The following countries were represented: Bulgaria, Croatia, the Czech Republic, Hungary, Israel, Kazakhstan, Poland, Romania, Russia, Serbia, Slovakia, Slovenia, and Ukraine. Experts attended an advisory board held in Bratislava, Slovakia, on 16 October 2019 to initiate long-term discussions that led to this review. The acromegaly experts from each country provided general and holistic information on the approaches from their country, details on the regional treatment center(s) and multidisciplinary team, access, reimbursement, and availability to approved treatments, as well as physician education, disease awareness, and patient advocacy. During discussions, the topics covered included disease prevalence and treatment patterns, current clinical challenges, and potential ways to optimize patient care across this region in the future (**Table 1**).

**Table 1:** Estimated prevalence of acromegaly, the median time to diagnosis, and number of specialist centers, by country.

Country (approximate total population*)	Estimated prevalence, per 1,000,000 inhabitants	Estimated current total number of patients diagnosed with acromegaly	Estimated median time to diagnosis, years	Initial presentation to which healthcare practitioners?	References
**Bulgaria (7.3 million)**	50	400	8	GPs, endocrinologists, gastroenterologists, gynecologists, dentists	
**Croatia (4.4 million)**	85	~300	7 (range: 4–9) Unpublished data from CRO-aCRO Registry	GPs, endocrinologists, neurosurgeons, ophthalmologists, dentists	
**Czech Republic (10.5 million)**	No data available	600	4	GPs, endocrinologists, internal medicine specialists, neurologists	
**Hungary (9.9 million)**	55–69	450	3–9	GPs, endocrinologists, neurosurgeons, internal medicine specialists	
**Israel (9.3 million)**	80	700	4–6	GPs, endocrinologists, neurosurgeons, orthopedic surgeons	
**Kazakhstan (17.1 million)**	40–70	Complete data not available	5–10	GPs, endocrinologists	
**Poland (38.1 million)**	70	2500	6	GPs, endocrinologists, internal medicine specialists	
**Romania (21.4 million)**	No data available	1000	4–7	GPs, endocrinologists, neurosurgeons, rheumatologists, pneumologists	
**Russia (146.2 million)**	23–90; varies by region	4300	6 (2–25)	GPs, endocrinologists, neurosurgeons, gynecologists	(5)
**Serbia^†^ (6.9 million)**	60	400	5–7	Neurosurgeons, neurologists, endocrinologists, cardiologists, radiologists	
**Slovakia (5.4 million)**	No data available	350	5	GPs, endocrinologists, neurologists	
**Slovenia (2.1 million)**	60	70	5–10	GPs, endocrinologists, dentists, ear/nose/throat specialists, plastic surgeons, neurosurgeons, pulmonologists, gynecologists	
**Ukraine (45.1 million)^‡^**	No data available	Complete data not available	9	Endocrinologists, neurosurgeons	(6, 7)

*Source: https://www.economist.com/node/21566456. Updated Nov 2012. Accessed 5 August 2021.

^†^Source: https://www.stat.gov.rs/en-us/oblasti/stanovnistvo/procene-stanovnistva/. Updated July 2021. Accessed 5 August 2021.

^ǂ^45.1 million demographic maximum. In 2019 an electronic census estimated that Ukraine’s population, excluding occupied territories to be 37.3 million.

Data from specialist centers in the region were used to estimate prevalence rates, ranging from 23 to 130 cases per million inhabitants (**Table 1**). This low prevalence with large-ranging differences within and between countries of the region may reflect the absence of official or mandatory national registries that are needed to produce reliable and freely available epidemiological data. Notably, the prevalence estimates shown in **Table 1** are typically based on the number of patients treated in specialist centers that may not cover the entire population of a given country or region. Therefore, the panel concluded the importance of a national registry and the need for initiating this process.

## Early Diagnosis and Comprehensive Approach

Owing to its diverse clinical presentation and the slow progression of symptoms that often overlap with those of other diseases, acromegaly is first suspected and eventually diagnosed by endocrinologists in the vast majority of cases (2, 4). After their initial primary care evaluation, patients with acromegaly often seek consultation in different disciplines for their symptoms, such as gynecologists for amenorrhea and fertility problems, ENT specialists for snoring, pulmonologists for obstructive sleep apnea, diabetologists for new-onset diabetes, and orthopedic surgeons for carpal tunnel syndrome. This time lag between the initial signs and symptoms and confirmed acromegaly diagnosis represents an important issue, and this varies somewhat by country across CEE, Israel, and Kazakhstan (**Table 1**). According to the pan-European database study, the median time from symptom onset to diagnosis was ten years in women and eight years in men (2, 4). It is noteworthy that this study showed a narrowing in the time gap between initial symptom presentation and diagnosis in recent years, which appears to be related to improved diagnostic modalities (4). **Table 1** shows the estimated median time to acromegaly diagnosis, similar to previously reported data (2, 4); however, a wide range (3-10 years) was observed across CEE, Israel, and Kazakhstan.

During the panel discussion, it was reported that in Romania, Russia, and Ukraine, some patients with pituitary tumors might be presented directly to neurosurgery without hormonal evaluation or presurgical endocrinological consultation. The panel agreed unanimously that this approach is misleading and emphasized that pre- and post-operative endocrinological evaluation is mandatory. The experts recommended that endocrinologists work closely with neurosurgeons and participate in neurosurgical meetings to increase awareness of acromegaly and its management, encouraging referrals to surgeons at pituitary tumor centers of excellence.

Given the range of signs and symptoms associated with acromegaly, the expert panel concluded that a crucial step for reducing the time to diagnosis is to improve disease awareness and its cluster of signs and symptoms among non-endocrinologist healthcare practitioners.

Additionally, it is important to adopt scoring tools such as ACROSCORE and ACRO-POLIS that have been developed to aid physicians in earlier identification of the disease and further reduce the delay to diagnosis and treatment by taking into account multiple signs and symptoms associated with acromegaly, such as colorectal polyps, carpal tunnel syndrome, spaced teeth, and thyroid hyperplasia (2, 8). A recent study by Antsiferov et al. (2021) demonstrated the effectiveness of selective screening for improving diagnosis and more accurately assessing the prevalence of acromegaly. Unfortunately, such tools have not yet been widely adopted in the region (9). For example, in Bulgaria, Russia, Croatia, and Slovenia, ACROSCORE is not currently used; in many cases, translation into local languages and raising awareness amongst physicians who encounter cases are planned or in progress to encourage adoption into clinical practice. Similar approaches with other tools would likely be beneficial, and it is expected that the implementation of screening and scoring tools will facilitate the identification of likely cases of acromegaly by general practitioners and non-endocrinology specialists.

## Comorbidities

Acromegaly is a chronic disease associated with severe complications and comorbidities that may persist despite biochemical control of the disease (10, 11). The severity of these complications is correlated with diagnostic delay and the presence of active acromegaly (1). These include cardiovascular and metabolic complications (5, 12–15), in addition to respiratory, intestinal, and skeletal disorders and particular neoplasms (5, 14, 16–19), all of which can impair patients’ quality of life and may contribute to increased mortality (10). Women of reproductive age may also experience menstrual cycle disorders that coincide with the initial onset of acromegaly symptoms (20). Data from Bulgaria and Poland showed that 95% of patients suffered from comorbidities, with other endocrine and metabolic diseases and cardiovascular disease being the most common (12, 21), co-occurring in 50% of patients (12). Of note, while the overall mortality in patients with acromegaly has decreased, the biochemical control of disease activity with normalized GH and IGF-I does not always correlate with clinical well-being (10). Findings from a Polish cohort suggest that acromegaly disease activity is correlated with metabolic comorbidities rather than disease duration (22). Therefore, managing comorbidities concomitantly with biochemical disease activity control is an important issue, although control of GH and age-related IGF-I levels remains the cornerstone of treatment (10).

Indeed, advances in the clinical management of the disease can go some way to addressing the issue of comorbidities, as demonstrated in a single-center study performed in Croatia (23). Data spanning three decades of acromegaly management in France indicate that the proportion of patients who achieve disease control with medical therapy has increased over time. In these patients, the incidence of comorbidities is low, with life expectancy close to that in the general population (24). Ongoing vigilance for comorbidities is required to maintain this progress and could be supported by screening for other uncommon “hidden” comorbidities. A recent study of 106 patients with acromegaly and matched healthy controls indicated a surprisingly high incidence of bone damage, sometimes asymptomatic, in patients with acromegaly. It is of particular interest that vertebral fracture was identified in 13 acromegalic patients, compared with 4 in the control group (25). Similarly, Mazziotti et al. (2013) demonstrated a significantly higher incidence of vertebral fractures among patients with prolonged active acromegaly duration compared with controls (26). A study from Slovakia recently reported that vertical fractures occur despite disease control and are best predicted by cortical volumetric bone mineral density (27). A study from Croatia has shown lower calcaneus quantitative ultrasound parameters and increased bone turnover in male patients with active acromegaly (28). Hepatic steatosis is another comorbidity that has recently been reported at relatively high rates in patients with acromegaly, though the studies included small numbers of patients (29, 30). Genetic markers that may help identify patients with increased risk from these comorbidities have been studied (30, 31).

The panel concluded that physician awareness of acromegaly-associated comorbidities must be improved, including asymptomatic “hidden” complications such as osteoporosis and vertebral fractures. In addition, the group emphasized the importance of concomitant management of these comorbidities in order to improve the quality of life and optimally control disease activity for patients with acromegaly.

## Use of Clinical Guidelines in the Region

In general, international guidelines are followed wherever possible as these tend to be more up-to-date than local guidelines. For example, a treatment algorithm from a recently published consensus statement from the 11^th^ Acromegaly Consensus Conference is widely referred to in clinical practice internationally, including in the countries considered in this review (32). One exception is Ukraine, where clinical practice aligns where possible with the Endocrine Society guidelines, depending on the availability of medications (33). Local guidelines are followed in countries where these are available and up-to-date, such as Poland and Russia (34, 35).

## Clinical Care: Variation Across the Region

Similar principles of best clinical practice are followed across the region, however, there are some minor differences in the specific treatment modalities as identified by the expert panel (**Table 2**). Moreover, the panel reported several factors that might influence treatment approaches from country-to-country, including availability, the proximity to neurosurgery and radiosurgery centers, and treatment reimbursement (**Table 3**).

**Table 2:** Different treatment modalities according to the panel experts from CEE, Israel, and Kazakhstan.

Country	First line	Second line	Third line	Fourth line	Fifth line	Notes
**Bulgaria**	Surgery	DA in patients with mild disease or first-generation SRL	Combination treatment with first-generation SRL and DA or PEGV or all three	PEGV (as monotherapy or combination with DA) or PAS-LAR (as monotherapy or combination therapy)		Radiosurgery is considered to be an option at every stage of treatment after failure of pharmacological therapy
**Croatia**	Surgery	First-generation SRL or DA in selected patients	First-generation SRL + PEGV or first-generation SRL + DA or PAS-LAR monotherapy or PEGV monotherapy (in SRL non-responders)	First-generation SRL+ PEGV + DA		Radiosurgery is considered to be an option at every stage of treatment
**Czech Republic**	Surgery	Gamma knife radiosurgery (Leksell gamma knife; linear accelerator) + pharmacotherapy	Cabergoline (in patients with mild disease activity) or First-generation SRL or first-generation SRL + cabergoline or PAS-LAR	PEGV or PEGV + first-generation SRL or PEGV + cabergoline		
**Hungary**	Surgery	DA (in patients with mild disease)	First-generation SRL or first-generation SRL + DA	SRL + PEGV or PEGV monotherapy or PAS-LAR monotherapy		Radiosurgery is considered to be an option at every stage of treatment
**Israel**	Surgery	First-generation SRL	First-generation SRL + PEGV ± cabergoline or PEGV or PAS-LAR			Radiosurgery is considered to be an option at every stage of treatment
**Kazakhstan**	Surgery	First-generation SRL	First-generation SRL + DA	Radiosurgery		
**Poland**	First-generation SRL (before surgery)	Surgery	First-generation SRL (as first pharmacotherapy after surgery)	PAS-LAR or PEGV (therapeutic program)	PEGV + first-generation SRL	Repeat surgery is considered at every stage of treatment. Stereotactic radiosurgery is considered when surgery has failed
**Romania**	Surgery	First-generation SRL	First-generation SRL + DA	First-generation SRL + PEGV, or PEGV monotherapy, PAS-LAR or radiosurgery		Repeat surgery is considered at every stage of treatment
**Russia**	Surgery (or first-generation SRL when total tumor removal is not possible or qualified neurosurgeon is not available)	First-generation SRL or cabergoline monotherapy (mild disease)	First-generation SRL + cabergoline	Radiosurgery or PEGV		
**Serbia**	Surgery	First-generation SRL	First-generation SRL + cabergoline	PAS-LAR or PEGV monotherapy or combination treatment		Radiosurgery is considered if pharmacological treatment is ineffective
**Slovakia**	Surgery (sometimes with debulking treatment using SRL)	First-generation SRL	First-generation SRL + PEGV	Gamma knife radiosurgery or second surgery		
**Slovenia**	Surgery	First-generation SRL	First-generation SRL + PEGV or PEGV monotherapy or PAS-LAR monotherapy	PAS-LAR + PEGV		Radiosurgery used in selected cases after initial surgery. Cabergoline used in patients with mild disease, either as monotherapy or in combination with first-generation SRLs or PEGV. PAS-LAR is typically used in young patients without diabetes, who show signs of continued tumor growth, experience persistent headaches, or are intolerant to PEGV.
**Ukraine**	Surgery	First-generation SRL or PEGV or radiosurgery	Repeat surgery or combined medical treatment	Repeat radiosurgery	Temozolomide for aggressive tumors	Cabergoline in patients with mild disease

DA, dopamine agonist; SRL, somatostatin receptor ligand; PAS-LAR, long-acting pasireotide; PEGV, pegvisomant.

**Table 3:** Multidisciplinary centers, treatment availability, and interdisciplinary pituitary boards according to the panel experts from CEE, Israel, and Kazakhstan.

Country (approximate total population*)	Number of multidisciplinary sites with on-site neurosurgery (A), endocrinology(B) and radiotherapy (C)	Estimated disease control rates	Availability/reimbursement of first-generation SRL	Availability/reimbursement of PEGV	Availability/reimbursement of PAS-LAR	Regular interdisciplinary pituitary boards at institutional (I), local (L), or regional level (R) and patient advocacy group
**Bulgaria** **(7.3 million)**	A 1 B 1 C 1	84.3%	Octreotide LAR monotherapy	PEGV monotherapy/ combination with SRL	PAS-LAR monotherapy/ combination with PEGV	I, L Pituitary Association, Association of Acromegaly Patients in Bulgaria
**Croatia** **(4.4 million)**	A 2 B 1 C 1	95% (unpublished data from Croatian acromegaly registry)	First-generation SRL monotherapy	PEGV monotherapy/ combination with SRL	PAS-LAR monotherapy/ combination with PEGV or cabergoline	I (at one center) No patient advocacy groups
**Czech Republic** **(10.5 million)**	A 7 B 5 C 5	Estimated surgical cure rate 50–60%, pharmacotherapy disease control in the majority	First-generation SRL monotherapy/ combination with cabergoline	PEGV monotherapy/ combination with SRL or cabergoline	Reimbursed after approval of health insurance company	I, L, and R No patient advocacy groups
**Hungary** **(9.9 million)**	A 4 B 5 C 4	75%	First-generation SRL monotherapy/ combination with cabergoline	PEGV monotherapy/ combination with SRL or DA	PAS-LAR monotherapy/ combination with PEGV	No patient advocacy groups
**Israel** **(9.3 million)**	A 7 B 6 C 6	87%	First-generation SRL monotherapy	PEGV monotherapy/ combination with SRL	PAS-LAR monotherapy	I, L and annual acromegaly patient day
**Kazakhstan** **(19.0 million)**	A 3 B 1 C 1	Estimated rate cured by surgery: 50%	First-generation SRL monotherapy	Not reimbursed	Not reimbursed	I
**Poland** **(38.1 million)**	A5 B12 C 2	75%	Therapy with first-generation SRL reimbursed	Available in medication program	Available in medication program	Annual acromegaly patient day Patient advocacy group
**Romania** **(19.2 million)**	A 3 B 3 C 1	52%	First-generation SRL monotherapy	PEGV monotherapy/ combination with SRL or DA	PAS-LAR monotherapy/ combination with PEGV or cabergoline	Monthly multidisciplinary meetings (L). Tumor board ad hoc meetings (L) Patient website (Romanian Society for Endocrinology) (I)
**Russia** **(146.2 million)**	A6 B10 C2	24–50%	First-generation SRL monotherapy/ combination with DA	Reimbursed	Not available	I, L, and R Velikan patient society
**Serbia^†^** **(6.9 million)**	A 2 B 2 C 2	~70%	First-generation SRL monotherapy/ combination with DA	PEGV reimbursed	PAS-LAR reimbursed	Acromegaly patient society (UPAK-Udruzenje pacijenata sa akromegalijom) https://upak.rs
**Slovakia** **(5.4 million)**	A 4 B 4 C 0	60–75% after TSS for microadenoma and 50% for macroadenoma. 75% in combination with medical therapy	First-generation SRL monotherapy	PEGV	PAS-LAR	
**Slovenia** **(2.1 million)**	A 1 B 2 C 0	80%	First-generation SRL monotherapy/ combination with DA	PEGV monotherapy/ combination with SRL or DA	PAS-LAR monotherapy/ combination with PEGV	Monthly (I) and biannual (R) multidisciplinary meetings/ Not established
**Ukraine** **(45.1million)^ǂ^**	A 1 B 1 C 4	Unknown	First-generation SRLs are partially reimbursed from regional budgets	PEGV is partially reimbursed from regional budgets	Available for administration, without reimbursement	Regular interdisciplinary pituitary boards at institutional (I), local (L) or (R) at meetings and conferences. NGO "Rare Diseases of Ukraine" (http://rarediseases.org.ua/)

*Source: https://www.economist.com/node/21566456. Updated Nov 2012. Accessed 05 August 2021.

^†^Source: https://www.stat.gov.rs/en-us/oblasti/stanovnistvo/procene-stanovnistva/. Updated July 2021. Accessed 5 Aug 2021.

^ǂ^45.1 million demographic maximum. In 2019 an electronic census estimated that Ukraine's population, excluding occupied territories to be 37.3 million.

DA, dopamine agonist; SRL, somatostatin receptor ligand; PAS-LAR, long-acting pasireotide.

Published reports from the region also indicate differences in the clinical treatment and management of patients with acromegaly, which may impact on patient outcomes. For example, a recent study reporting data from a Romanian tertiary care center disclosed that improvements to disease control had been attributed to changes in reimbursement for second-line pharmacological treatment options as well as increased referrals of pituitary surgery to specialist centers (36). Notably, in Poland and in some regions of Russia, where the availability of expert neurosurgeons is limited, the use of presurgical treatment such as somatostatin receptor ligands (SRLs) are more common (34). Cumbersome applications for reimbursement may also limit options for pharmacological treatment in many countries in the region, as is the case with long-acting pasireotide (PAS-LAR) and pegvisomant (PEGV) (37). Considering that pharmacological treatment represents a pivotal modality in managing uncontrolled acromegaly postoperatively, these limitations to optimal management are likely to have unfavorable outcomes and might be less cost-effective in the long term (38). However, data on the cost-effectiveness of medical therapies are relatively limited (39).

The panel concluded that treatment should be according to acceptable worldwide guidelines and that surgery should be the first-line treatment modality for operable acromegalic patients.

## Key Clinical Considerations

According to the guidelines, the neurosurgical approach represents first-line modality of treatment, and this is common practice in Bulgaria, Croatia, the Czech Republic, Hungary, Israel, Kazakhstan, Romania, Serbia, Slovenia, Ukraine, and in some Russian regions where qualified pituitary surgeons are available (Moscow and surrounding regions such as Sankt-Petersburg, Tatarstan). For patients with uncontrolled postoperative acromegaly, the panel discussed some differences in pharmacological treatment between the 2020 Pituitary Society updates to the acromegaly management guidelines and everyday clinical practice in the region (40).

According to the consensus statement, treatment with first-generation long-acting SRLs such as octreotide LAR or lanreotide autogel, with dose escalation for partial responders, represents first-line pharmacological treatment (17). However, it is well known that the proportion of patients who achieve a complete response is suboptimal. According to a recently published retrospective study from a tertiary medical center in Bulgaria, 50% (59/118) of the patients achieved disease control using treatment with a single-agent SRL (21). The expert panel agreed that increasing the dose of an SRL was thought to be ineffective in most patients and is not always attempted in clinical practice considering that only a small proportion of partial responders are likely to achieve a complete response with a higher dose. This was also observed in a 24-week prospective, randomized, open-label study of high-dose vs. high-frequency lanreotide autogel in partial responders after >6 months of SLR treatment. Treatment with either regimen achieved less than one-third normalization of IGF-I, with no significant difference in efficacy between high-dose vs. high-frequency (41). Similar data were reported in a prospective, randomized study using PAS-LAR vs. octreotide LAR. Despite up-titration being permitted in either treatment arm, fewer than 30% achieved biochemical control at 12 months by dose up-titration (42).

The option of multiple treatment modalities for second-line pharmacological treatment such as PAS-LAR and PEGV as monotherapy or in combination with first-generation SRLs allows for treatment individualization and disease control optimization. Key points to be considered when choosing treatment should include the extent of residual tumor, impaired glucose tolerance or new-onset diabetes, and whether the patient has experienced any side effects related to treatment.

PAS-LAR has been shown to provide sustained efficacy in a small proportion of patients treated previously with maximum doses of first-generation SRLs (43). In a Phase IIIb, open-label study of PAS-LAR, most adverse events were related to hyperglycemia. While almost all patients had diabetes or pre-diabetes at baseline, 63% of patients required antidiabetic treatment during the study, compared with only 25% of patients at baseline (43). As a result of PAS-LAR-induced hyperglycemia, some countries such as Serbia, Slovenia, and Croatia profile patients to determine who may benefit from second-line treatment such as PAS-LAR and PEGV. Furthermore, strategies to mitigate and monitor PAS-LAR-induced hyperglycemia have been published recently (44). In Romania, PAS-LAR is considered a second-line option, glucose levels are monitored regularly, antidiabetic treatment is initiated in the case of new-onset diabetes and treatment with PAS-LAR can be switched to PEGV monotherapy in the presence of persistent hyperglycemia. PAS-LAR treatment is not considered in patients with uncontrolled diabetes in Israel, Poland, and Hungary. Owing to concerns about side effects of PAS-LAR, some clinicians avoid using PAS-LAR in patients who have not achieved a response with first-generation SRLs, instead recommending a combination of first-generation SRLs and PEGV or PEGV monotherapy. It is also noteworthy that PAS-LAR was considered an optional modality of treatment for patients with uncontrolled acromegaly who experience persistent headaches (45). The experts note that treatment availability should be considered, for example, PAS-LAR is not yet available in Russia, Kazakhstan, or Ukraine.

In contrast to SRLs, PEGV appears to improve glucose metabolism, as reported in a recently published meta-analysis that included 18 prospective studies. This meta-analysis showed that PEGV significantly decreased fasting plasma glucose (FPG) and glycated hemoglobin (HbA_1c_) and that its effect on FPG was independent of its effect on IGF-I levels (46). However, when PEGV was used in combination with an SRL, the overall effect on glucose metabolism appeared to be neutral (46). Given the observational data supporting its long-term efficacy, PEGV is a key pharmacological option in this treatment setting (47). Nonetheless, careful clinical monitoring and proactive management – including dose adjustment – are needed to optimize disease control; for example, a recently published meta-analysis that demonstrated the effectiveness of PEGV monotherapy in real-world studies to be 71.7% (64.0-78.4%; 95% CI), which is not as high as may be expected from the results of interventional studies (48). Another consideration is whether PEGV could promote pituitary tumor growth, given its unique mechanism of action. Published evidence from a long-term observational study has been reassuring (47). However, treatment decisions should always take the size and aggressiveness of the adenoma, and its response to previous treatment, into account (49). This is reflected in treatment approaches in many countries in the region, where PEGV is not used as monotherapy to treat patients with large or growing tumors.

Finally, another consideration in first- and second-line pharmacological therapy is cabergoline, which is used in responsive patients with mild disease activity, either as monotherapy or in combination with first-generation SRLs or PEGV.

The rationale for combined treatment as second-line pharmacological therapy is that the addition of another modality of treatment with a different mechanism of action is more likely to show an additive effect than dose escalation of the ongoing treatment or switching compound altogether – especially for patients showing a partial response to first-line pharmacological treatment. This is supported by a recently published network meta-analysis of simulated trials generated by an algorithm, which indicated that combination therapy with PEGV and SRL was the most effective medical treatment option overall (50). Indeed, findings from the non-interventional ACROSTUDY show that the proportion of patients receiving combination treatment increased from 20% in 2003 to 54% in 2012 (51), with the trend likely continuing as pharmacological treatment options increase and become more widely reimbursed.

The role of radiotherapy and radiosurgery in patients who have not achieved biochemical control of acromegaly varies by location, reflecting differences in access to specialist centers and waiting times across CEE, Israel, and Kazakhstan. However, published data from other countries indicate that increased access across the region in question would have considerable potential to improve patient outcomes. For example, in a retrospective study of 352 patients in the German Acromegaly Registry, approximately three-quarters of patients were in remission or had controlled disease ten years after fractionated radiotherapy (FRT) or stereotactic radiosurgery (SRS). However, care is needed to avoid hypopituitarism. In this study, the rate of pituitary insufficiency was significantly higher after FRT (52). Another retrospective study including 371 patients across ten centers, including some in the Czech Republic, supports the efficacy of SRS in persistent or recurrent disease, showing an actuarial durable remission rate of 59% at ten years (53). The use of radiosurgery in the management of acromegaly also permits patients to reduce and eventually stop pharmacotherapy in the long term; the median time to normalization of IGF-I in one study of radiosurgery was 54 months (54). This is likely to have clear benefits for patients, as well as pharmacoeconomic benefits.

## Testing at Baseline and Follow-Up

After primary surgery, MRI scans to assess tumor volume are performed as early as 12 hours after surgery in some specialist centers, but typically at around three months, providing a baseline for future reference. After this, imaging is carried out at tailored time intervals based on tumor status, GH, and IGF-I response.

GH and IGF-I testing are the cornerstones of acromegaly diagnosis and subsequent monitoring of disease activity (55). Improvements have been made to assays for each of these, making them more sensitive and reliable on the whole. However, reference ranges and clinical thresholds still vary between assays, for example, in Poland, active acromegaly is diagnosed when IGF-1 levels are above 1.0 × ULN and GH suppression is not below 1.0 μg/L using oral glucose tolerance tests (OGTTs) or below 0.4 mg/L using ultrasensitive GH assays (34). In Croatia and Slovenia, a GH cut-off of 0.4 µg/L is also used for diagnosis. Standardization of when and how to test GH and IGF-I levels during diagnosis and follow-up would substantially improve the clinical management of acromegaly, providing information of consistent quality on which to base treatment decisions.

IGF-I tests are generally done every six months after surgery in patients with well-controlled disease. IGF-I assay results can vary with the assay used and the laboratory that performs it (56). Therefore, these findings support using the same assay throughout one patient’s diagnosis and follow-up. To aid consistent interpretation of IGF-I test outputs, many specialist centers develop their database and reference range for adults by sex and age group, based on consistent use of a specific assay. Tests should be performed exclusively at the reference laboratory, and training and education of laboratory specialists are also recommended to enhance the consistency of test outputs.

A similar principle applies to GH testing, in which the need for consistency also extends to reference ranges. GH nadir concentrations during OGTTs using sensitive GH assays were recently reviewed, and normal values were lower than the thresholds specified in previous acromegaly guidelines and variable according to body mass index, sex, and oral contraceptive use.

## Disease Control Across CEE, Israel, and Kazakhstan

Despite different treatment modalities, disease control remains a challenge for many patients treated in this region. Data from a Russian registry reported that the proportions of patients undergoing neurosurgical treatment increased from 36% to 50% in 2012–2019, while the proportion of patients undergoing radiation therapy decreased from 17.7% to 0.8% in the same period (57). This study also reported remission rates of 40% after neurosurgery and 29% after medical treatment as first-line therapy (p<0.01). Retrospective data from 147 patients treated in Romania who had been assessed at least once at a tertiary referral center, showed 29% disease control – defined as random serum GH <1 μg/L and age-normalized serum IGF-I. Of these, more than 90% had undergone surgery, and ~80% were receiving pharmacological treatment with SRLs (58). More recent published studies have shown that the proportion of patients with biochemical control is already being improved: another study performed in Romania demonstrated that 46.7% of the participants achieved disease control (34); a retrospective study from 191 patients of a tertiary center in Bulgaria demonstrated 84% disease control, with the most common pharmacotherapies used after surgery being single-agent long-acting SLRs (38%) or combination therapy with PEGV and an SLR (12%). As would be expected, treatment with combination therapy appeared to be more common in patients who had longer disease duration (21). The potential for achieving high response rates with carefully tailored pharmacotherapy is further highlighted by a recent retrospective study in Israel, which reported good biochemical control in 87% of a cohort of 87 patients with active acromegaly who underwent medical treatment, most after surgery failure (59). The above-reported data demonstrated an improvement in the management of acromegaly and disease control activity across CEE, Israel, and Kazakhstan.

The expert panel concluded that to achieve better disease control, there is a need for systematic evaluation of disease activity based on multiple parameters, including the use of defined tools such as the Acromegaly Disease Activity Tool (ACRODAT^®^), which includes IGF-I level, tumor status, symptoms, comorbidities, and quality of life (60). Another such multidimensional instrument is SAGIT^®^, which covers a combination of clinical and biochemical features, including signs and symptoms (S), associated comorbidities (A), GH levels (G), IGF-I levels (I), and tumor profile (T) (61, 62). Acromegaly-specific patient-reported outcomes, such as those included in the Acro-TSQ score, could also provide valuable insights to guide tailored patient care at specific points in the treatment algorithm (63). Unfortunately, these tools, like diagnostic tools such as ACROSCORE, have not been widely adopted in the region, likely again due to lack of translations into local languages and consequent low awareness.

Patient-centered, multidisciplinary care should be the foundation that supports improvements to diagnosis, follow-up, management of comorbidities, and patient quality of life. Multidisciplinary care is already underway across CEE, Israel, and Kazakhstan. Typically, the pituitary multidisciplinary team includes an endocrinologist, neurosurgeon, pathologist, and radiologist. In the Czech Republic and Croatia, this multidisciplinary team also includes a specialist in stereotactic surgery; in Serbia, it includes a geneticist. Provision of care *via* specialist centers varies widely across the region, with some countries having more specialist pituitary centers per capita of the population than others (**Table 1**). Finally, systematic data collection in national or regional registries would support identifying and exchanging of best practices and better screening for and management of comorbidities. Registries could also potentially include specific reporting requirements for test results, facilitating widespread standardization and allowing comparison between patients. Data from registries could also be used to inform and support reimbursement requests or policies.

## Conclusions

The management of acromegaly across CEE, Israel, and Kazakhstan has been advanced in the last decade. However, there is a need for further improvement in this field, including early diagnosis by increasing disease awareness among non-endocrinologist healthcare practitioners; standardized tools for disease activity evaluation; the systematic gathering of data in national registries for precise data reporting, including prevalence and other parameters. Furthermore, the panel revealed the importance of multidisciplinary disease management and treatment accessibility for surgical and pharmacologic therapies. The continued exchange of best practices in these areas can support optimizing patient care across the region.

## Author Contributions

All authors contributed to the advisory board discussions, critically reviewed all drafts of the manuscript and approved the final draft of the manuscript.

## Funding

Pfizer supported the face-to-face advisory board meeting.

## Conflict of Interest

The authors declare competing interests as follows: MB reports personal fees and non-financial support from Novartis; grants, personal fees and non-financial support from Ipsen; personal fees, non-financial support and other from Pfizer; and personal fees from Recordati outside the submitted work. MD reports personal fees from Pfizer and personal fees from Novartis outside the submitted work. MG reports personal fees from Pfizer and personal fees from Novartis outside the submitted work. VH reports personal fees and non-financial support from Pfizer and Ipsen outside the submitted work. II reports lectures’ fees from Pfizer and Ipsen outside the submitted work.

DK reports personal fees from Pfizer outside the submitted work. TK reports personal fees from Pfizer and personal fees from Novartis outside the submitted work. MK reports personal fees from Pfizer outside the submitted work. CP reports personal fees from Pfizer, Novartis and Ipsen outside the submitted work. SV reports personal fees from Pfizer outside the submitted work. RG reports employment with Pfizer. SP reports employment with Pfizer. DS reports employment with Pfizer. IS reports personal fees and other from Pfizer; personal fees and other from Medison Pharma; and grants, personal fees and other from Novartis outside the submitted work.

The remaining authors declare that the research was conducted in the absence of any commercial or financial relationships that could be construed as a potential conflict of interest.

This project received funding from Pfizer. The funder had the following involvement with the study: supported the face-to-face advisory board meeting and funded medical writing and editorial assistance.

## Publisher’s Note

All claims expressed in this article are solely those of the authors and do not necessarily represent those of their affiliated organizations, or those of the publisher, the editors and the reviewers. Any product that may be evaluated in this article, or claim that may be made by its manufacturer, is not guaranteed or endorsed by the publisher.
